# What every intensivist needs to know about subsyndromal delirium in the intensive care unit

**DOI:** 10.5935/0103-507X.20200004

**Published:** 2020

**Authors:** Rodrigo Bernardo Serafim, Maria Carolina Paulino, Pedro Povoa

**Affiliations:** 1 Instituto D’Or de Pesquisa e Ensino - Rio de Janeiro (RJ), Brazil.; 2 Hospital Copa D’Or - Rio de Janeiro (RJ), Brazil.; 3 Instituto de Doenças do Tórax, Hospital Universitário Clementino Fraga Filho, Universidade Federal do Rio de Janeiro - Rio de Janeiro (RJ), Brazil.; 4 Unidade Polivalente de Terapia Intensiva, Hospital de São Francisco Xavier, Centro Hospitalar de Lisboa Ocidental - Lisboa, Portugal.; 5 NOVA Medical School, Universidade Nova de Lisboa - Lisboa, Portugal.; 6 Epidemiology Center and Clinical Epidemiology Research Unit, Odense University Hospital - Odense, Denmark.

## Introduction

Several studies have described the negative outcomes associated with *delirium* in the short or long term,^(1,2)^ but not every form of *delirium* has the same prognosis. The duration and severity of *delirium* have been found to be the main factors associated with worse outcomes.^(3,4)^ In fact, a very short *delirium* duration seems to have little impact on the mortality rates of patients admitted to the intensive care unit (ICU).^(3)^ Despite advances in the recognition of *delirium*, there is still a large number of patients who present acute cognitive dysfunction during the ICU stay but still do not meet the criteria for the diagnosis of *delirium*.^(5)^ These patients were classified as having a condition known as subsyndromal *delirium* (SSD).^(6,7)^ Subsyndromal *delirium* has been commonly reported as an intermediate stage between delirium and normal mental status, but there is little knowledge about its pathophysiology and epidemiology.

## How can subsyndromal delirium be diagnosed?

There is no published consensus on the definitions of subclinical forms of *delirium,* and there is no specifically developed tool for the diagnosis of SSD. The Diagnostic and Statistical Manual of Mental Disorders, 5^th^ edition, used the term “attenuated *delirium* syndrome” to describe a condition very similar to SSD, but without specific diagnostic criteria, and it has been under discussion whether the entity SSD should be added as a subcategory of *delirium*, in parallel with another new category, mild neurocognitive disorder.^(8)^ Studies that have evaluated SSD used preexisting tools for *delirium* diagnosis. The most frequently employed *delirium* screening tools consider a diagnosis of SSD when the Intensive Care Delirium Screening Checklist (ICDSC) score is 1 - 3 out of 8 or when the Confusion Assessment Method (CAM) or CAM-ICU score was positive on 1 or 2 items out of 4.^(5)^

## What is the prevalence of subsyndromal delirium in the intensive care unit?

Considering the available studies, the prevalence of SSD in the ICU is near 45% but can vary from 13% to 52% according to the studies.^(5)^ The different forms of evaluation of SSD and the different populations studied contribute to this high variation in the reported prevalence rates (Table 1). In fact, risk factors for SSD are the same for *delirium*, and high-risk populations (e.g., elderly, mechanically ventilated) have a higher prevalence.^(5)^ We also believe that the prevalence of SSD can be underestimated. When SSD is considered as a slight mental status change evaluated with an intermittent screening assessment, it can be easily underrecognized.

**Table 1:** Characteristics of the studies on subsyndromal delirium

Reference	Patients enrolled (n)	Type of patients	Delirium screening tool	Patients with subsyndromal delirium,	Nº of patients with delirium,	ICU LOS in subsyndromal delirium group	ICU LOS in delirium group	ICU LOS in non-delirium group
				n (%)	n (%)	Days (SD)	Days (SD)	Days (SD)
Breu et al.^(7)^	467	Cardiac surgery	ICDSC	158 (39)	54 (12)	2.0 (2.0)	3.0 (3.75)*^1^	2.0 (2.0)*^1^
Brummel et al.^(9)^	821	Medical/surgical	CAM-ICU	702 (86)	NA	NA	NA	NA
Al-Qadheeb et al.^(10)^	763	Mechanically ventilated	ICDSC	481 (63)	282 (37)			
Hakim et al.^(11)^	177	Cardiac surgery in an elderly population	ICDSC	101 (57)	NA	NA	NA	NA
Azuma et al.^(12)^	70	Medical/surgical	ICDSC	22 (31.4)	NA	NA	NA	NA
Yamada et al.^(13)^	380	Medical/surgical	ICDSC	129 (33.9)	60 (15.8)	2.0 (0.5)	NA	2.0 (0.5)
Sanson et al.^(14)^	199	Cardiac surgery	ICDSC	66 (30)	68 (31)	NA	NA	NA
Boettger et al.^(15)^	289	Medical/surgical	DSM-IV-TR	36 (13)	86 (30)	NA	NA	NA
Li et al.^(16)^	38	Surgical	CAM	13 (34)	7 (18)	NA	NA	NA
Tan et al.^(17)^	53	Cardiac surgery	CAM	18 (34)	12 (23)	NA	NA	NA
Oiumet et al.^(18)^	537	Medical/surgery	ICDSC	179 (33)	189 (35)	5.2 (4.9)*^1^	10.8 (11.3)*^2^	2.5 (2.1)*^1,2^

ICU - intensive care unit; LOS - length of stay; SD - means standard deviation; ICDSC - Intensive Care Delirium Screening Checklist; DSM-IV-TR - Diagnostic and Statistical Manual of Mental Disorders Fourth Edition text review; CAM - Confusion Assessment Method; NA - data not available. P values are:

* 1,2 p < 0,01, +p = 0.49.

## What is the impact of subsyndromal delirium occurrence in the intensive care unit?

Although studies in non-ICU patients have shown that SSD is associated with a higher risk of death, the same was not found in critically ill patients. A systematic review of non-ICU older patients described that SSD was associated with an increase in hospital length of stay, post-discharge mortality and functional decline.^(6)^ However, despite the apparent importance of SSD in non-ICU settings, studies in ICU populations have not described a consistent increase in the risk of death.^(5)^ These differences can be explained by the high burden of *delirium* non-modifiable risk factors, frequently present early at the onset of critical illness, which contribute to the occurrence of *delirium* without a prodromal phase or SSD in the ICU. This may also indicate that the occurrence of SSD (a condition of lower severity compared with rapidly reversible *delirium*) may not be sufficient to increase death.

In a recent meta-analysis including six studies and 2630 ICU patients, SSD was diagnosed in 36% of patients. Subsyndromal *delirium* was associated with an increase in the duration of hospital stay (odds ratio 0.31; 95% confidence intervals - 95%CI 0.12 - 0.51, p = 0.002; I2 = 34%) but was not associated with mortality (hazard ratio 0.97; 95%CI 0.61 - 1.55; p = 0.90).^(5)^ Moreover, in another recent publication that included 821 ICU patients, SSD was described in 86% of the patients, and it was an independent predictor of institutionalization. Patients who presented SSD for 5 days or more had a greater chance of being institutionalized after discharge compared with those who presented SSD for only 1.5 days (adjusted odds ratio 4.2; 95%CI 1.8 - 9; p = 0.007).^(9)^

The impact of SSD on mechanical ventilation was also evaluated in only one study, which described a non-clinically relevant increase in weaning time (10.0 ± 8.0 *versus* 11.0 ± 10.75 hours, p < 0.01) in SSD patients compared with those with normal mental status.^(10)^

## Treatment of subsyndromal delirium and progression to delirium

There is no evidence that the pharmacological or nonpharmacological treatment of SSD can change its trajectory or outcomes. Studies investigating the use of antipsychotic drugs to prevent the progression of SSD to *delirium* have shown controversial results.^(10,11)^ In one of these studies, the use of haloperidol in patients with SSD reduced the number of hours the patient was agitated but did not influence the proportion of episodes of *delirium* or the duration of the *delirium*.^(10)^ In another study, the administration of risperidone to elderly patients who suffered SSD after cardiac surgery with extracorporeal circulation was associated with a lower incidence of *delirium*. No study has evaluated nonpharmacological strategies to prevent the progression of SSD to delirium in the ICU.^(5,11^

Despite an unclear benefit in the treatment of SSD, we believe that SSD monitoring is important for identifying patients at risk for delirium and for improving complementary measures such as sleep control or pharmacological review. Moreover, SSD can be the first sign of a mental dysfunction or an underlying disease in critically ill patients.

## Future directions

It remains unclear whether SSD represents an early stage of full *delirium*, an independent diagnosis, or simply a description for an array of symptoms with no major clinical consequence. To date, studies on SSD in the ICU have focused on small and heterogeneous populations. Future studies should focus on the evaluation of larger populations of critically ill patients employing standardized definitions, describing the cognitive trajectory of SSD, or using new quantitative scales such as the CAM Short Form^(12)^ and the CAM-ICU-7 (a version of the CAM in which responses are based on a 7-point scale)^(19)^ that seem to be more aligned with the proposed diagnosis of SSD or with the graduation of cognitive dysfunction.

## Conclusion

Subsyndromal delirium is a frequent condition in intensive care unit patients. The occurrence of subsyndromal *delirium* is associated with a longer intensive care unit and hospital stay but not with an increase in mortality. Monitoring subsyndromal *delirium* can help intensivists to identify patients at risk of delirium or patients with a worse prognosis. Further studies are needed for a better understanding of subsyndromal *delirium* relevance in intensive care unit patients as well as its treatment.
